# A Bumblebee-Inspired Spatial Memory Navigation Framework for Robotic Odor Source Localization

**DOI:** 10.3390/biomimetics11050350

**Published:** 2026-05-18

**Authors:** Tianyi Xu, Yizhu Guo, Zhigang Wu, Jianing Wu

**Affiliations:** 1 School of Aeronautics and Astronautics, Sun Yat-sen University, Shenzhen 518107, China; xuty29@mail2.sysu.edu.cn; 2 Beijing Institute of Spacecraft System Engineering, Beijing 100094, China; luckbamboo7@126.com; 3 School of Advanced Manufacturing, Sun Yat-sen University, Shenzhen 518107, China

**Keywords:** bumblebee, odor source localization, spatial memory, navigation

## Abstract

Odor source localization in turbulent environments remains a major challenge for autonomous robots, as odor plumes are highly intermittent, spatially fragmented, and often lack stable concentration gradients. Here, we propose a bio-inspired navigation framework that translates key principles of bumblebee olfactory cognition into robotic decision-making. First, classical conditioning and olfactorily triggered spatial memory experiments demonstrated that bumblebees could form robust odor memories and that training frequency is positively correlated with both proboscis extension response retention and spatial directional preference. Based on these biological findings, a bio-inspired navigation framework, termed Bio-Nav, is constructed by integrating a Partially Observable Markov Decision Process, a Hidden Markov Model, short-term memory, long-term directional reference memory, fuzzy inference, and value iteration. High-fidelity two-dimensional turbulent simulations show that the proposed algorithm substantially outperforms moth-inspired search, Infotaxis, and standard POMDP-based navigation. In 100 Monte Carlo trials, Bio-Nav achieved a success rate of 96.0%, an average of 20.3 search steps, an average path length of 155.1 cm, and a path-to-straight-line distance ratio of 1.6. Even under strong turbulence, the success rate remained above 91%. These results indicate that memory–perception coupling, inspired by bumblebee navigation, provides an effective and robust strategy for odor source localization in complex turbulent environments, offering a generalizable principle for bio-inspired robotic search under uncertainty.

## 1. Introduction

In a complex physical world characterized by high uncertainty, accurate autonomous localization from weak and sparse signals remains a frontier problem in robotics and automation [1]. Odor source localization (OSL) is particularly relevant to hazardous source inspection in confined spaces, deep-space exploration, autonomous plume tracking by micro aerial vehicles, and post-disaster search-and-rescue missions [2,3,4]. Unlike light or sound, however, odor molecules are transported primarily by turbulent fluid motion and therefore form fragmented, intermittent plumes rather than smooth spatial gradients. Conventional gradient-following chemotaxis or purely upwind anemotaxis can become trapped in local optima under high-Reynolds-number turbulence or degenerate into inefficient random wandering once odor cues are lost. These limitations have motivated increasing interest in biologically inspired approaches that can operate robustly under intermittent sensing conditions [5,6].

In nature, many insects exhibit remarkable capabilities in locating odor sources within highly turbulent environments. Among insects, hymenopterans—especially bumblebees [7], honeybees [8,9], and wasps—display remarkable olfactory navigation and spatial cognition. In broad natural environments with highly complex airflow, these insects can precisely locate food sources and subsequently return to the nest using weak floral odor cues [10]. Behavioral studies based on the Proboscis Extension Response (PER) and related conditioning paradigms indicate that this outstanding navigational ability is supported not only by instantaneous perception but also by strong coupling between olfactory learning and memory [11]. A broad body of work further suggests that bumblebee olfactory memory spans multiple time scales [12,13]. Short-term memory helps insects respond to nearby turbulent odor patches, whereas longer-lasting memory supports directional persistence and reuse of previously rewarding spatial information when signals become intermittent [14,15]. This property is especially relevant to robotic search in turbulent environments, where brief signal loss is common. Taken together, these biological findings suggest that introducing bumblebee olfactory cognition and short- and long-term memory mechanisms into robotic navigation algorithms may provide an effective route to overcoming the bottlenecks of odor source localization in turbulent environments [16,17].

Despite these biological insights, translating such mechanisms into robotic systems remains challenging [18]. In turbulent environments, sensor observations are sparse, noisy, and often reduced to binary plume encounter or non-encounter events. Accordingly, odor source localization is fundamentally a sequential decision-making problem in a partially observable environment [19,20]. The combination of a Hidden Markov Model (HMM) and a Partially Observable Markov Decision Process (POMDP) provides a natural foundation for this class of problems [21]. Within this framework, the HMM models the time-evolving odor plume and wind field, while the POMDP maintains a belief state over possible source locations and supports action selection under uncertainty. As a consequence, these methods often exhibit unstable trajectories, inefficient exploration, or failure under prolonged signal loss. In particular, conventional probabilistic models lack mechanisms to preserve directional continuity or to exploit accumulated experience when sensory input becomes unreliable.

To address this limitation, this paper proposes a novel bio-inspired navigation algorithm that integrates the POMDP-HMM framework with bumblebee olfactory cognition [22]. The central idea is to retain probabilistic inference as the backbone for handling turbulence-induced uncertainty while introducing a higher-level spatial memory module with both short-term and long-term components. Short-term memory is used to discourage repeated local revisits and thereby improve global exploration efficiency, whereas long-term directional memory preserves attraction toward historically informative regions and stabilizes the search direction when instantaneous cues become unreliable. This design bridges purely mathematical estimation and persistent, experience-dependent biological behavior.

Situated at the intersection of bionics and robotic control engineering, this study aims to solve robust odor source localization in complex turbulent environments. The work includes three components—biological experiments, mathematical modeling, and high-fidelity simulations—and makes the following contributions.

1. A bio-inspired navigation architecture integrating probabilistic modeling with biologically grounded short-term and long-term memory is constructed.
2. Behavioral experiments quantify how repeated odor–reward training strengthens both proboscis extension response retention and odor-triggered spatial preference in bumblebees.
3. Comparative simulations show that the proposed framework improves search success, path efficiency, and robustness under intermittent plume conditions.

The remainder of this paper is organized as follows. Section 2 presents the biological experiments, Section 3 establishes the integrated navigation framework, Section 4 reports comparative simulation studies under different flow field conditions, Section 5 discusses the main findings, Section 6 summarizes the limitations and engineering migration feasibility, and Section 7 concludes the paper. The navigation framework proposed in this paper is based on three layers: the probabilistic estimation layer, the memory stabilization layer, and the adaptive fusion and policy-generation layer. It is hereinafter referred to as “Bio-Nav”. The nomenclature list of variables is shown in Table A1.

## 2. Materials and Methods

To support the design of Bio-Nav at the behavioral level, the biological experiments in this study were not treated as independent observations of insect behavior. Instead, they were organized to provide direct evidence for the two functional requirements of the navigation algorithm: (i) odor–reward association must be learnable and strength-dependent, (ii) learned odor cues must be transferable into persistent spatial preference. Accordingly, this design allowed the behavioral evidence to map directly onto the memory formation, memory dominance, and adaptive fusion mechanisms later implemented in Bio-Nav.

In the context of robotic odor source localization, it is insufficient to show only that bumblebees can detect odor and respond to reward. What is more important is whether odor experience can be accumulated, whether it can be transformed into spatially structured guidance, whether the resulting memory weakens over time, and whether previously stored experience can compete with or complement current online odor input. These questions determine whether a navigation algorithm should include memory modules, how memory strength should be updated, and how online sensory evidence should be fused with stored internal states. For this reason, the experiments reported in this section were designed as a behavioral evidence chain for the later three-layer architecture of Bio-Nav.

### 2.1. Bumblebee Sample Preparation

Adult worker bumblebees were collected at Sun Yat-sen University (Shenzhen, China) (Latitude 22.46° N, Longitude 113.54° E) and reared in hives. The samples were adult worker bumblebees of *Bombus terrestris*, with a body mass of 200 ± 100 mg and an estimated age range of 20–201360 days after emergence. Temperature and humidity were maintained at 25 °C and 50%, respectively. Bumblebee behavior outside the hive was recorded using a digital camera. All experiments were conducted within 24 h of capturing bees from the hive. We confirmed that no special permits were required for the experimental location/activities. Experiments were conducted from 15 June to 15 July 2025.

### 2.2. Classical Conditioning Experiment

**Figure 1:**
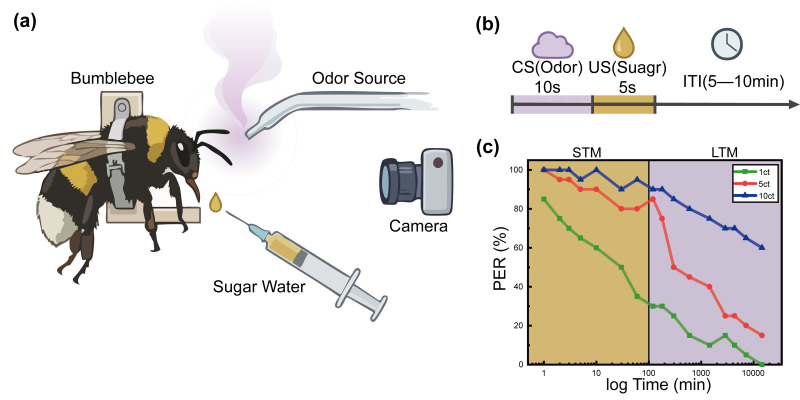
Classical Conditioning Experiment. (**a**) Experimental device diagram. (**b**) Experimental flowchart. (**c**) Proboscis extension response.

The classical conditioning experiment was designed to determine whether bumblebees could establish a stable odor–reward association and whether the strength of this association depended on training frequency [23]. A modified centrifuge tube was used as a restraint device to gently immobilize the body while allowing free movement of the head, antennae, and proboscis. Carnation essential oil (concentration:20%, manufacturer: Nefertari) was delivered as the conditioned stimulus, and sugar water was used as the unconditioned reward (Figure 1a) [24]. In each training trial, the bee was first exposed to the odor for 10 s, after which sugar water was presented. The 10 s odor-exposure period and the 5–10 min inter-trial interval were selected according to standardized PER-conditioning procedures reported in previous insect olfactory-learning studies and were further confirmed in preliminary trials as sufficient to evoke antennal sampling without causing apparent fatigue or habituation [25]. Following the resting interval, the same procedure was repeated (Figure 1b).

A total of 60 adult worker bumblebees with similar body size were randomly assigned to three groups (20 individuals per group). Group I received 1 training trial, Group II received 5 training trials, and Group III received 10 training trials. After training, the proboscis extension response (PER) to the same odor was tested at multiple post-training time points, and the temporal change in response probability was analyzed [25].

The results showed that PER retention increased monotonically with training frequency. Bumblebees trained for 5 or 10 trials displayed both higher response probability and longer retention than bees trained for a single trial. Across groups, response probability decreased gradually with increasing post-training delay, but the rate of decline was substantially smaller in the higher-training groups. Statistical comparison indicated significant effects of training frequency and retention interval on PER expression. In Group I, a certain proportion of responses could be observed within a short period of time, but the retention time was relatively short (Figure 1c). As the delay time increased, the response rate decreased rapidly. The response of Group II to the same odor stimulus was more stable, and a higher PER incidence rate could be maintained at multiple time points. Group III demonstrated the most obvious advantage in memory retention, not only having the highest overall response probability but also maintaining a recognizable response level even after a longer delay. It can be seen from this that the more times the bumblebee is trained, the more stable its olfactory memory becomes, and the longer its behavioral response to olfactory cues last [25].

These findings indicate that odor memory in bumblebees is not a transient reflex-like reaction, but a trainable internal state whose behavioral expression depends on prior experience. In other words, repeated odor–reward pairing increases the behavioral salience of the associated cue. For Bio-Nav, this result provides the biological basis for assigning accumulated behavioral value to odor-associated states rather than treating each odor encounter as a memoryless instantaneous event.

### 2.3. Olfactorily Triggered Spatial Memory Experiment

**Figure 2:**
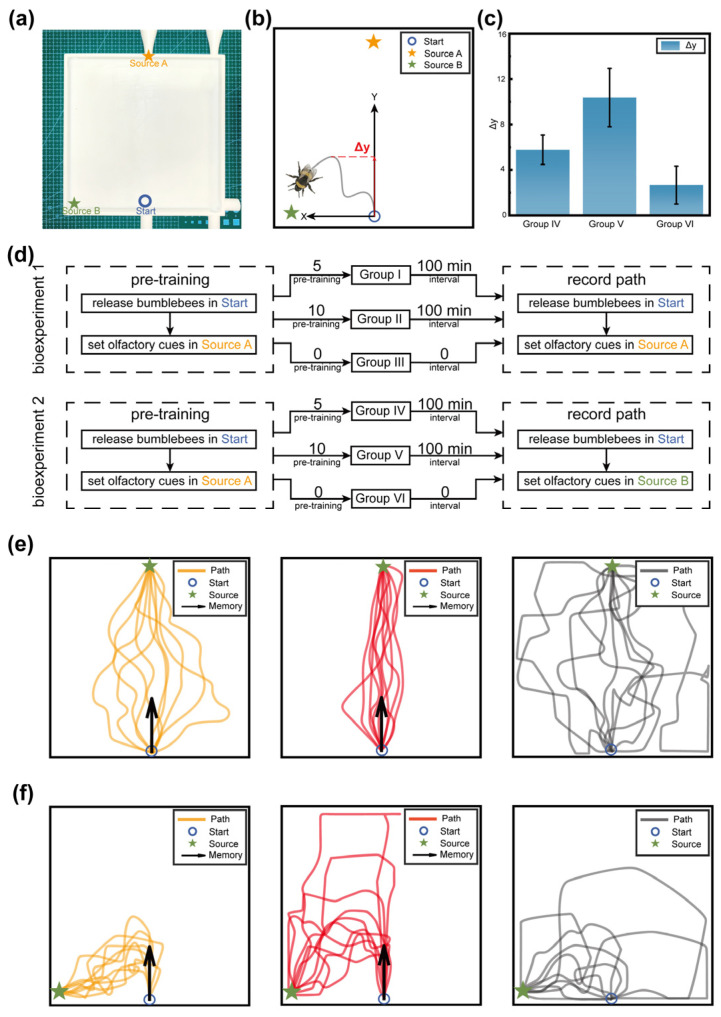
Olfactorily Triggered Spatial Memory Experiment. (**a**) Experimental device for olfactorily triggered spatial memory experiment. (**b**) Definition diagram of Δy. (**c**) Three groups of bumblebees Δy statistical graph. (**d**) The flowchart of olfactorily triggered spatial memory experiment. (**e**) The path of olfactorily triggered spatial memory experiment 1. (**f**) The path of olfactorily triggered spatial memory experiment 2.

To test whether learned odor cues could guide free movement in space rather than merely triggering reflexive responses in restrained individuals [26], we performed a spatial memory experiment using a square arena of 200 mm × 200 mm with a 30 mm entrance channel at the bottom. Odor-release ports were located at the top (Source A) and at the lower-left sidewall (Source B), while bumblebee release positions were located at the middle of the bottom edge (Start) (Figure 2a). A total of 60 bumblebees were divided into six groups for two bio-experiments.

In bio-experiment 1, 30 bumblebees were randomly divided into Groups I–III. Groups I and II received 5 and 10 pre-training trials, respectively, in which bumblebees were released from Start and rewarded at Source A. Group III received no pre-training. After pre-training, all bees were released again from Start under unchanged conditions, and their trajectories were recorded individually. In bio-experiment 2, another 30 bumblebees were divided into Groups IV–VI. Groups IV and V received 5 and 10 pre-training trials, respectively, from Start to Source A, while Group VI received no pre-training. However, during the testing phase, the experimental conditions changed. The odor and sugar water rewards were placed at Source B. The path of every bumblebee was recorded under the new condition (Figure 2d).

In bio-experiment 1, most bumblebees eventually located Source A, but clear between-group differences were observed in path organization. Pre-trained bees exhibited more concentrated and efficient trajectories than untrained controls, and the effect was stronger in Group II than in Group I. These results indicate that repeated odor–space pairing improves path convergence and reduces blind exploration (Figure 2e). In bio-experiment 2, all groups eventually located Source B, but Groups IV and V showed a marked tendency during the early phase of the trial to deviate toward the previously trained direction (toward Source A), even though no odor was released there during testing. By contrast, Group VI moved more directly toward the actual source and showed no stable directional bias (Figure 2f).

To quantify this effect, the odor information perceived at the entrance was defined as an X-direction cue, whereas the spatial bias established during pre-training was defined as an orthogonal Y-direction cue. The Y-direction displacement Δy was therefore used as an indicator of odor-triggered spatial memory strength (Figure 2b). Both the mean and maximum Y-direction displacements were larger in Groups IV and V than in Group VI, and Group V exceeded Group IV (Figure 2c).

These findings demonstrate that familiar odor cues can trigger a persistent directional spatial bias rather than simply eliciting local attraction [7,27]. For Bio-Nav, this result directly supports the introduction of long-term directional reference memory, because the behavioral evidence suggests that previously rewarding odor encounters are encoded as structured spatial preferences that continue to influence future navigation.

### 2.4. Memory Decay Experiment

**Figure 3:**
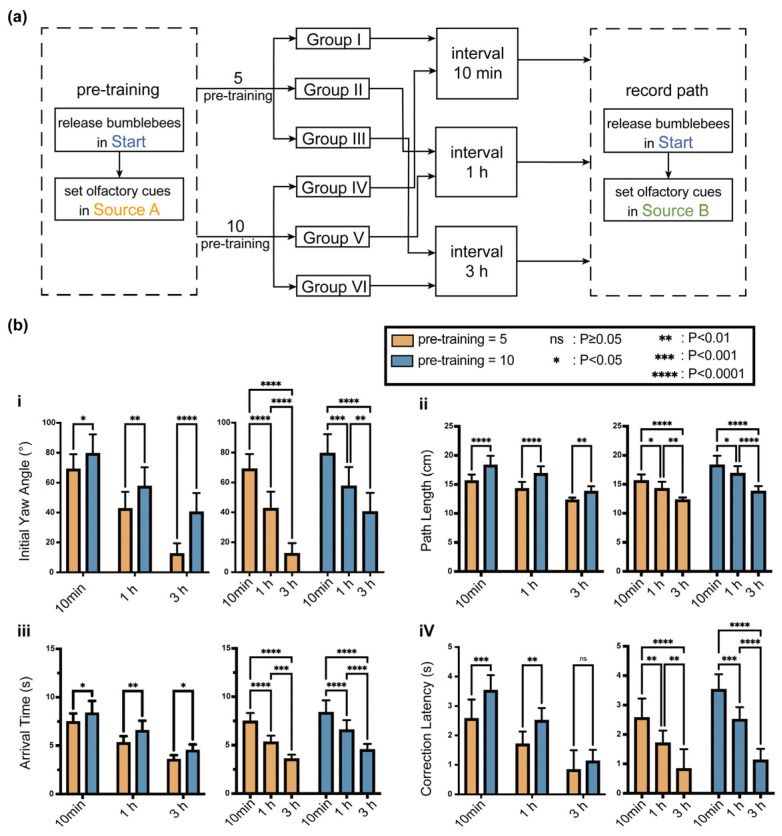
Memory decay and reactivation experiment for odor-triggered spatial memory in bumblebees. (**a**) Experimental workflow of the memory-decay experiment. Bumblebees were first trained to associate the odor cue and reward with Source A under different training intensities and then tested after different retention intervals. During the test phase, Source A was no longer rewarded, while Source B provided the current reward. This design was used to examine whether previously learned odor–space memory decayed over time and whether the residual memory trace still affected the early movement direction under a new reward condition. (**b**) Interaction effects of training intensity and retention interval on four behavioral parameters. (**i**) Initial yaw angle, defined as the angular deviation between the initial movement direction and the direction toward the current rewarded source, reflects the early directional bias induced by the previous memory. (**ii**) Path length quantifies the redundancy of the navigation trajectory. (**iii**) Arrival time measures the time required for the individual to reach the target region near Source B. (**iv**) Correction latency represents the time required for the bee to correct its initial deviation from the previously memorized direction toward the current rewarded source. The results indicate that stronger training produced a more persistent directional memory effect, whereas longer retention intervals weakened this memory-driven bias. Statistical significance is indicated as follows: n.s. denotes no significant difference, *p* ≥ 0.05; * denotes *p* < 0.05; ** denotes *p* < 0.01; *** denotes *p* < 0.001; and **** denotes *p* < 0.0001.

To investigate whether odor-triggered spatial memory decays over time and whether weakened memory traces can be reactivated by familiar odor cues, a memory decay and reactivation experiment was designed based on the previous spatial memory paradigm. The group labels in this experiment were reset and do not refer to the Groups I–VI used in Section 2.3. A total of 60 bumblebees were divided into six new groups according to a two-factor design: training intensity (5 or 10 pre-training trials from Start to Source A) and retention interval (10 min, 1 h, or 3 h). In the test phase, all bees were released from Start and rewarded at Source B, while Source A remained unrewarded (Figure 3a).

Figure 3b further illustrates how the behavioral expression of odor-triggered spatial memory depends jointly on training intensity and retention interval. A larger initial yaw angle and longer correction latency indicate stronger interference from the previously rewarded direction, whereas a shorter path length and arrival time indicate more efficient adaptation to the current rewarded source. Therefore, these four parameters jointly describe the dynamic transition from memory-dominated navigation to current-cue-dominated correction.

Two-factor ANOVA was used to evaluate the effects of training intensity and retention interval. The results showed main effects of both factors on initial yaw angle, path length, arrival time, and correction latency. Interaction effects were also observed, indicating that memory decay was jointly modulated by prior training strength and retention duration (Figure 3b).

### 2.5. Cue-Conflict Decision Experiment

**Figure 4:**
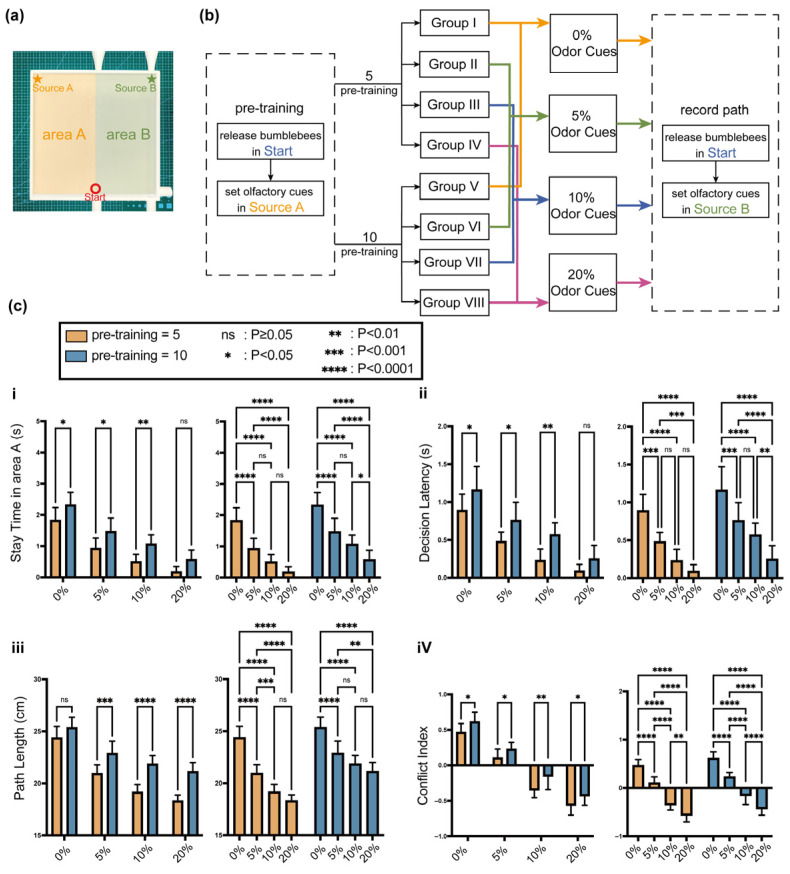
Cue-conflict decision experiment for evaluating the arbitration between stored spatial memory and current odor evidence. (**a**) Experimental arena used for the cue-conflict decision experiment. Bumblebees were first trained to associate the odor cue and reward with Source A, thereby establishing an odor-triggered spatial memory toward the previously rewarded location. During the test phase, Source A no longer provided odor or reward, whereas Source B provided the current odor cue and sugar-water reward at different odor concentrations. (**b**) Experimental workflow of the cue-conflict paradigm. The experiment independently manipulated memory strength through training intensity and current sensory reliability through odor concentration at Source B. This design allowed us to examine how bumblebees dynamically weighted historical spatial memory against online odor information when the two cues were inconsistent. (**c**) Interaction effects of training intensity and odor concentration on four decision-related behavioral parameters. (**i**) Stay time in area A reflects the degree of dependence on the previously rewarded memory location. (**ii**) Decision latency measures the time required for the bee to form a stable movement direction under conflicting cues. (**iii**) Path length evaluates the overall navigation efficiency during conflict resolution. (**iv**) Conflict index quantifies the relative tendency to move toward the old memory location versus the current odor source during the early trajectory segment. The results show that bumblebee navigation under cue conflict was not governed by either memory or current odor alone, but by dynamic arbitration between the two. Statistical significance is indicated as follows: n.s. denotes no significant difference, *p* ≥ 0.05; * denotes *p* < 0.05; ** denotes *p* < 0.01; *** denotes *p* < 0.001; and **** denotes *p* < 0.0001.

To determine how bumblebees resolve conflict between previously learned spatial preference and current online odor evidence, a cue-conflict decision experiment was conducted. Eighty bumblebees were divided into 8 groups, and they were first trained from Start to Source A under repeated odor–reward pairing, thereby establishing a stable odor-linked directional memory in the arena (Figure 4a). During the testing phase, every bumblebee was released from Start. Source A no longer provided sugar water rewards or emitted odors; Source B, according to the group, provided 0%, 5%, 10%, or 20% odor cues while also offering sugar water rewards (Figure 4b). The purpose of this experimental design was to be able to separately control “memory strength” and “current perception reliability”, thereby observing the weights of the two in the bumblebee’s navigation decisions.

Behavioral indicators mainly include: First, the Stay Time in area A, which is used to characterize the individual’s dependence on the old memory location. Second, the Decision Latency, which is the time experienced from release to the formation of a stable orientation. Third, the Path Length, which is used to comprehensively evaluate the navigation efficiency under conflicting conditions. Fourth, the Conflict Index, which is used to describe the individual’s weighing process in the conflict cues, is the difference between the cumulative displacement towards the old memory location and the cumulative displacement towards the actual odor source in the first segment of the trajectory, after normalization, to obtain the indicator. After statistical analysis, interaction effects could be observed in these four parameters (Figure 4c). The four behavioral variables in Figure 4 were selected to characterize different aspects of cue-conflict resolution. Stay time in area A and conflict index mainly reflect the influence of the old memory trace, whereas decision latency and path length reflect the behavioral cost of resolving inconsistency between memory and current odor evidence. The interaction between training intensity and odor concentration therefore provides direct behavioral support for the adaptive fusion mechanism later implemented in Bio-Nav.

Behavioral results showed that the decision pattern was not all-or-none. Many individuals displayed an early trajectory component biased toward the previously learned direction, followed by gradual correction toward the currently rewarded source. The final success rate remained high, indicating that the new odor cue was still behaviorally effective, but the early trajectory structure revealed that the stored memory trace strongly influenced initial decision-making. This result provides the strongest behavioral rationale for the adaptive fusion mechanism in Bio-Nav. If navigation were driven only by current odor evidence, the early bias toward the old direction would not appear. If navigation were driven only by memory, bees would fail to correct their trajectories toward the actual source. The path suggests that bumblebee navigation relies on dynamic weighting between online evidence and stored experience, which is exactly the principle implemented by the adaptive fusion layer of the algorithm.

### 2.6. Summary of Biological Experiments

Taken together, the biological experiments demonstrate that bumblebee odor-guided navigation cannot be explained by instantaneous sensing alone. Classical conditioning shows that odor-associated value is learnable and training-dependent. The olfactorily triggered spatial memory experiment shows that this learned value becomes directionally organized and continues to influence later navigation. The memory decay experiment demonstrates that the internal representation is dynamic rather than static, and that weakened memory traces can be re-expressed under familiar sensory cues. Finally, the cue-conflict experiment reveals that navigation emerges from context-dependent arbitration between stored experience and current online odor information.

These results collectively motivate the three-layer design of Bio-Nav. The probabilistic estimation layer accounts for the uncertainty of the external sensory environment, the memory stabilization layer accounts for the persistence and updating of internal experience, and the adaptive fusion layer accounts for the dynamic coordination between the two.

## 3. Bio-Inspired Olfactory Navigation Framework

Bio-Nav is organized as a three-layer navigation architecture rather than as a simple combination of probabilistic modeling and bio-inspired heuristics. The first layer, probabilistic estimation, infers the most likely source location and the most likely plume distribution under partial observability and turbulent transport. The second layer, memory stabilization, preserves directional continuity and suppresses inefficient local revisits when sensory evidence becomes sparse or intermittent. The third layer, adaptive fusion, dynamically arbitrates between online probabilistic evidence and stored spatial experience to generate the reward landscape used for action selection.

This organization is motivated directly by the biological experiments presented in Section 2. The classical conditioning experiment shows that odor-associated value can be learned and strengthened through repetition. The olfactorily triggered spatial memory experiment shows that learned odor cues become directionally organized and continue to bias later navigation. The memory decay experiment shows that this memory is dynamic and time dependent. The cue-conflict experiment further shows that stored experience and current odor input are not used independently but are weighted adaptively according to the current behavioral context. Bio-Nav translates these behavioral principles into a unified engineering framework for odor source localization in turbulent environments.

### 3.1. Probabilistic Estimation Layer

#### 3.1.1. Search-Space Discretization

The odor source localization task is defined in a two-dimensional rectangular search domain Ω, which is discretized into a regular grid to facilitate probabilistic state estimation and action selection. Suppose the domain is divided into m rows and n columns, yielding a total of

(1) M=m×n

grid cells. Each cell is treated as a candidate hidden source state. Let

(2) $$C=[C_{1},C_{2},\ldots ,C_{M}]$$

denote the discrete state space. The geometric center of cell $C_{i}$ is denoted by $C_{i}=(x_{i},y_{i})$.

To map between the one-dimensional state index i and the two-dimensional grid coordinates (f,g), the following bijection is defined as

(3) $$f\left(i\right)=rem\left(i-1,m\right)+1\;\;g(i)=int(i-1,m)+1$$

where $rem\left(n,m\right)$ represents the remainder of n divided by m, and int(n,m) denotes the largest integer not exceeding n/m. This discretization provides the spatial support for all subsequent maps in Bio-Nav, including the source-belief map, the plume-distribution map, and the memory map.

#### 3.1.2. Advection–Diffusion Plume Model

In turbulent environments, odor does not form a smooth and continuous concentration gradient. Instead, it appears as a fragmented and intermittent collection of filaments transported by both mean flow and stochastic turbulent diffusion. The motion of an odor filament at time t can be modeled as

(4) $$\dot{X}(t)=V(X,t)+N(t)$$

where $V(X,t)=(v_{x},v_{y})$ is the deterministic wind velocity and N(t) is a zero-mean Gaussian perturbation representing turbulent fluctuation. The covariance of N(t) reflects turbulence intensity.

If a filament is released from source located at $X_{s}$ at time $t_{l}$, the position of this filament $X\left(t_{l},t_{k}\right)$ at a subsequent time $t_{k}\left(t_{k}>t_{l}\right)$ is

(5) $$X(t_{l},t_{k})=X_{s}(t_{l})+\int_{t_{l}}^{t_{k}}\;V(X(\tau ),\tau )d\tau +\int_{t_{l}}^{t_{k}}\;N(\tau )d\tau$$

Accordingly, the conditional probability density function $p_{ij}(t_{l},t_{k})$—which quantifies the likelihood that a plume detected by the robot at cell $C_{j}$ at time $t_{k}$ originated from a source at cell $C_{i},$ released at time $t_{l}$—can be approximated as:

(6) $$p_{ij}(t_{l},t_{k})\approx \frac{L_{x}L_{y}}{2\pi (t_{k}-t_{l})\sigma_{x}\sigma_{y}}exp\left(-\frac{\left(x_{j}-x_{i}-s_{x}{)}^{2}\right.}{2(t_{k}-t_{l})\sigma_{x}^{2}}-\frac{\left(y_{j}-y_{t}-s_{y}{)}^{2}\right.}{2(t_{k}-t_{l})\sigma_{y}^{2}}\right)$$

Here, $s_{x}$ and $s_{y}$ are the wind-induced displacement components derived from the integral of historical anemometer data. This physical model forms the basis for both the source-belief update and the plume prediction process.

#### 3.1.3. POMDP-Based Source-Belief Update

Because the robot cannot directly observe the true source location, odor source localization is formulated as a Partially Observable Markov Decision Process (POMDP) [28]. The hidden state is the true source cell, while the robot receives only incomplete and noisy observations from local odor sensing.

Let the **Observation Space (**Ω**)** be

(7) $$\Omega =\{d,\bar{d}\}$$

where d denotes plume detection at time t and $\bar{d}$ denotes no detection.

**Observation Probability (**O**)** defines the probability $P(o^{\prime }|s_{i},a)$. If the robot at time $t_{k}$ fails to detect a plume (event $\bar{d}$), it implies that no filaments released from the source (at $C_{i}$), from $t_{0}$ to $t_{k},$ have reached the current location. Defining $\kappa_{ij}(t_{0},t_{k})$ as the joint probability of “missed detection” given a source at $C_{i}$, the observation model is:

(8) $$P(o^{\prime }|s_{i},a)=\left\{\begin{array}{l} 1-\kappa_{ij}(t_{0},t_{k}),\;\;\;\;\;\;\;\;if\;o^{\prime }=d\;(Detection)\\ \kappa_{ij}(t_{0},t_{k}),\;\;\;\;\;\;\;\;\;\;\;\;if\;o^{\prime }=\bar{d}\;(No\;Detection)\;\;\;\; \end{array}\right.$$

The **Source-Belief State** vector represents the belief map and the core advantage of this method is the recursive Bayesian update of the belief state $b_{t}(s)$. $b_{t}(s)$ can be defined as:

(9) $$b_{t}(s_{i})=\frac{P(o_{t}|s_{i})\cdot b_{t-1}(s_{i})}{\sum_{j=1}^{M}\;P(o_{t}|s_{j})\cdot b_{t-1}(s_{j})}$$

where $b_{t}(s_{i})$ is the posterior probability that the source is in cell $s_{i}$.

This belief map answers the question: **Where is the source most likely to be?** It is therefore the exploitation-oriented component of the probabilistic estimation layer.

#### 3.1.4. HMM-Based Plume Distribution Prediction

While the POMDP estimates “where the source is”, the robot also needs to know “where the plume is likely to be” to recover the trail if the signal is lost. We employ a Hidden Markov Model (HMM) to predict the dynamic propagation of the plume.

Let $\alpha_{j}(t_{0},t_{k})$ be the probability that cell $C_{j}$ contains a detectable filament at time $t_{k}$. The propagation of filaments is modeled as a Markov process with a state transition matrix A(t), where element $a_{nm}(t)$ represents the probability of a filament moving from $C_{n}$ to $C_{m}$ under current wind conditions.

The Plume-Distribution Map $\alpha (t_{k})$ is calculated by superimposing the distributions of filaments released at all historical timesteps $t_{q}$. Utilizing the forward algorithm of HMM, the map is updated recursively:

(10) $$\Psi (t_{0},t_{k})=\frac{1}{k+1}\left[I+k\Psi \left(t_{0},t_{k-1}\right)A\left(t_{k-1}\right)\right]\;\;\alpha (t_{k})=b\cdot \Psi (t_{0},t_{k})$$

where b is the current source probability vector. This model leverages historical wind data to predict curved or segmented plume shapes, providing a crucial “re-acquisition” guide for the robot during the exploration phase.

The HMM-based plume-distribution map answers the question: **Where is odor most likely to be encountered next?** It is therefore the exploration-oriented component of the probabilistic estimation layer.

#### 3.1.5. Functional Role of the Probabilistic Estimation Layer

Together, the source-belief map $b_{t}(s_{i})$ and the plume-distribution map $\alpha (t_{k})$ provide a statistically grounded estimate of the external environment under uncertainty. However, because both maps remain strongly dependent on incoming observations, their directional stability weakens when plume detection becomes rare, intermittent, or temporarily absent. This limitation motivates the introduction of the second layer, memory stabilization.

### 3.2. Memory Stabilization Layer

#### 3.2.1. Biological Motivation

The biological experiments showed that bumblebee navigation is influenced not only by current odor input but also by previously acquired odor-linked spatial experience [11,14]. This influence is not static: it strengthens with repetition, decays with time, and can be reactivated by familiar cues. Bio-Nav translates this behavioral principle into a memory stabilization layer consisting of short-term memory (STM) and long-term memory (LTM) [21].

STM is designed to reduce inefficient local revisits, which corresponds to an engineering analog of inhibition of return. LTM is designed to preserve the directional significance of previously informative odor encounters, thereby maintaining global search continuity under sparse sensory conditions.

#### 3.2.2. Short-Term Memory

Let $M_{t}^{STM}(C_{i})$ denote the short-term memory intensity of cell $C_{i}$ at time t. When the robot visits cell $C_{i}$, its STM value is activated to a maximum level $M_{max}^{STM}$. Thereafter, it decays exponentially:

(11) $$M_{t}^{STM}(C_{i})=M_{max}^{STM}\cdot exp\left(-\frac{t-t_{i}^{visit}}{\tau_{STM}}\right)$$

where $t_{i}^{visit}$ is the most recent visit time of cell $C_{i}$, and $\tau_{STM}$ is the STM decay constant.

Because STM represents recently explored locations, it is used as a repulsive term in decision-making. High STM values indicate that a region was recently sampled and should therefore be temporarily deprioritized. Functionally, STM suppresses oscillatory trajectories and improves local search efficiency.

#### 3.2.3. Long-Term Directional Reference Memory

Let $M_{t}^{LTM}(C_{i})$ denote the long-term memory intensity of cell $C_{i}$. LTM is updated when a strong odor event is detected. Unlike STM, which is strictly local and short-lived, LTM spreads the reinforcement toward the upwind region, reflecting the directional bias observed in the behavioral experiments.

If the robot detects a high-value odor event at cell $C_{i}$, the long-term memory update is defined as

(12) $$M_{t}^{LTM}(C_{i})=max\left[M_{t-1}^{LTM}(C_{i}),\alpha \cdot exp\left(-\frac{∥C_{i}-C_{k}-d_{up}{∥}^{2}}{2\sigma_{m}^{2}}\right)\right]$$

where α is the reinforcement strength, $d_{up}$ is the upwind directional offset, and $\sigma_{m}$ controls the spatial spread of the reinforced region.

This formulation ensures that previously informative odor encounters continue to exert directional influence even when current observations are weak or absent. Functionally, LTM acts as a global directional anchor.

#### 3.2.4. Memory Decay and Reactivation

Because biological memory is dynamic, both STM and LTM must evolve with time. STM decays rapidly by definition. LTM also decays, but at a slower rate:

(13) $$M_{t}^{LTM}(C_{i})=\lambda_{m}M_{t-1}^{LTM}(C_{i}),0<\lambda_{m}<1$$

where $\lambda_{m}$ is the long-term memory retention coefficient.

To model reminder-cue effects observed in the reactivation experiment, LTM can be refreshed when the current odor observation matches previously reinforced odor conditions:

(14) $$M_{t}^{LTM}(C_{i})\leftarrow M_{t}^{LTM}(C_{i})+\beta \;\phi_{t}(C_{i})$$

where β is the reactivation gain and $\phi_{t}(s_{i})$ denotes the current odor-triggered reactivation signal.

#### 3.2.5. Composite Memory Map

The composite memory map $m_{t}$ combines the repulsive STM component and the attractive LTM component:

(15) $$m_{t}(C_{i})=w_{L}M_{t}^{LTM}(C_{i})-w_{S}M_{t}^{STM}(C_{i})$$

where $w_{L}$ and $w_{S}$ are non-negative coefficients controlling the relative influence of the two components.

After normalization, $m_{t}$ provides a stabilization field that indicates both where the robot should continue searching and where it should avoid revisiting. In this way, the memory stabilization layer complements the probabilistic estimation layer by preserving behavioral continuity when online sensory evidence becomes unreliable.

### 3.3. Adaptive Fusion and Policy Generation

Neither probabilistic estimation nor spatial memory is universally sufficient for navigation in turbulent environments. When odor observations are reliable and frequent, the robot should rely more strongly on the probabilistic source belief. When plume contact has been lost, however, the system should depend more heavily on plume prediction and stored directional memory. Therefore, Bio-Nav does not use fixed-weight integration. Instead, it performs adaptive fusion according to the current search context.

This design is consistent with the cue-conflict decision experiment, which showed that bumblebees do not follow either online odor evidence or stored spatial memory exclusively. Rather, they display context-dependent behavioral arbitration between the two [22].

#### 3.3.1. Fuzzy Inference Controller

To implement this arbitration mechanism, Bio-Nav uses a fuzzy inference system (FIS) [29]. The controller takes three inputs and generates one output variable:

• Input 1: Sensed Plume Concentration (ρ). Reflects the richness of information at the current location. Fuzzy sets: Low (L), Medium (M), High (H).
• Input 2: Time Since Last Detection ($\delta_{T}$). Reflects the urgency of the search state. Fuzzy sets: Short (Sh), Average (Av), Long (Lo).
• Input 3: Local Memory Strength (μ). Reflects the historical value of the current vicinity. Fuzzy sets: Weak (W), Medium (M), Strong (S).
• Output: Fusion Weight (λ). A coefficient λ∈[0,1] regulating the relative contribution of online probabilistic evidence and memory-based stabilization. Fuzzy sets: Very Small (VS), Small (S), Middle (MI), Large (L), Very Large (VL).

A rule base of 27 fuzzy rules is constructed based on biological heuristics. Representative rules include:

Rule A: (Surging Behavior): IF Concentration is High (ρ is H) AND Time Since Detection is Short ($\delta_{T}$ is Sh), THEN the robot is likely in the plume center. The confidence in the Source Map should be maximized. λ is Very Large (VL).

Rule B: (Revisiting Behavior): IF Concentration is Low (ρ is L) BUT Memory is Strong (μ is S), THEN the robot has lost the plume but is in a high-value zone. It should maintain a moderate focus on the source to prevent excessive drifting. λ is Middle (MI).

Rule C: (Wide-Area Exploration): IF Time Since Detection is Long ($\delta_{T}$ is Lo) AND Memory is Weak (μ is W), THEN the robot is lost. It must rely on the Plume-Distribution Map (HMM) or explore new areas. λ is Small (S).

**Figure A1:**
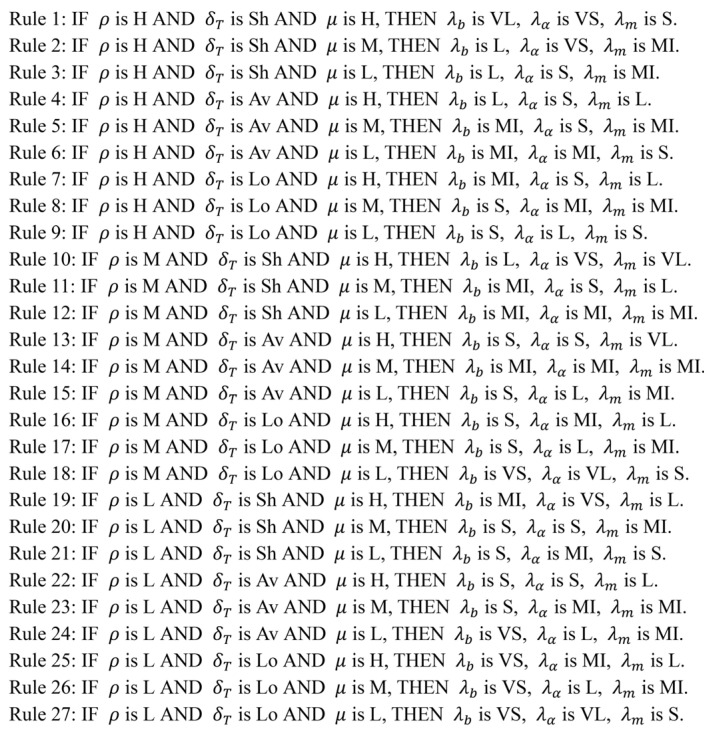
Complete 27-rule fuzzy inference table used in Bio-Nav.

The complete 27 fuzzy rules are provided in Appendix A, Figure A1.

#### 3.3.2. Dynamic Reward Construction

The adaptive fusion coefficient is then used to construct the immediate reward map [30]:

(16) $$R(C_{i})=\lambda \cdot \frac{b(C_{i})}{max(b)}+(1-\lambda )\cdot \frac{\alpha (C_{i})}{max(\alpha )}+\gamma_{m}\cdot m_{t}(C_{i})$$

where $b(C_{i})$ is the POMDP source belief, $\alpha (C_{i})$ is the HMM plume prediction, $m_{t}(C_{i})$ is the composite memory map, and $\gamma_{m}$ is the memory-scaling factor.

This reward map answers the central decision question of Bio-Nav: How should online evidence and stored experience jointly determine the attractiveness of each candidate action region?

#### 3.3.3. Value-Iteration-Based Action Selection

Once the dynamic reward map is obtained, action selection is performed using value iteration. Let $V(C_{i})$ denote the value function of cell $C_{i}$. Based on the Bellman optimality equation [31], the value function of a cell $C_{i}$ can be calculated by

(17) $$V(C_{i})=\underset{a\in A}{max}\;(R(s,a)+\gamma V(a))$$

where γ∈(0,1) is the discount factor, A is the set of 8-neighbor movement actions.

Importantly, value iteration is not treated here as an independent module parallel to probabilistic estimation or memory. Rather, it is the policy solver operating on the reward landscape generated by adaptive fusion. In this sense, adaptive fusion and value iteration together constitute the final decision layer of Bio-Nav.

The Bellman equation is repeatedly applied to update the value function V over the entire map until the value difference Δ between two adjacent iterations becomes smaller than the predefined threshold ϵ, indicating convergence.

Policy generation: the action that maximizes R(s,a)+γV(a) is selected as the current optimal policy $\pi^{*}$. The robot then executes this action, moves to the next position, and repeats the above process. At each time step, this algorithm generates a vector field pointing toward the globally optimal region. The robot only needs to execute the optimal action $\pi^{*}$ corresponding to its current grid cell, thereby achieving autonomous navigation throughout the whole process from plume discovery, to plume tracking, and finally, to source confirmation. In this way, the globally optimal path generated by the robot not only points toward the currently most probable source location but also accounts for plume re-acquisition and traversal of high-value memory regions.

### 3.4. Interpretation of the Three-Layer Architecture

In summary, Bio-Nav can be understood as a layered architecture in which each layer solves a different problem in odor-guided navigation.

The **probabilistic estimation layer** determines what the external environment currently suggests under uncertainty. It uses the POMDP source-belief map and the HMM plume-distribution map to estimate both source likelihood and plume reacquisition opportunity.

The **memory stabilization layer** preserves navigation continuity when online evidence becomes sparse, intermittent, or contradictory. By combining short-term repulsion from recently explored regions and long-term attraction toward historically valuable upwind areas, it prevents the robot from either oscillating locally or drifting aimlessly under signal loss.

The **adaptive fusion layer** determines how online evidence and stored experience should be weighted at each time step. By generating a context-dependent reward map and solving the corresponding action policy through value iteration, it allows Bio-Nav to switch smoothly between exploitation, plume reacquisition, and memory-guided persistence.

**Figure 5:**
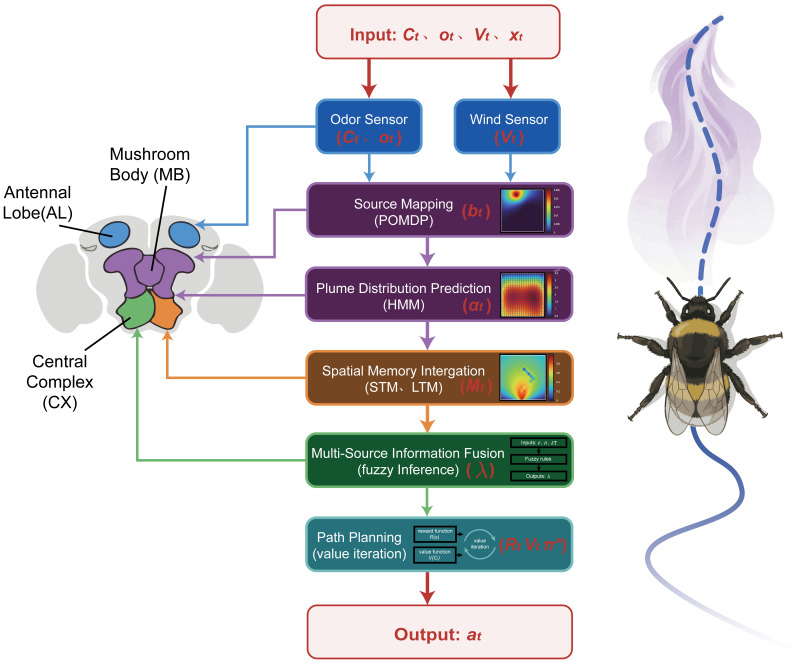
Flowchart of the Bio-Inspired Olfactory Navigation Algorithm with explicit sensor inputs, internal data flow, and action output.

To clarify the complete information flow of Bio-Nav, Figure 5 explicitly labels the sensor inputs, intermediate maps, and final action output. At each time step, the odor sensor provides local concentration and plume-encounter information, while the wind-speed sensor provides the local airflow vector for plume-transport prediction. These two sensory streams are not used independently; instead, they are jointly transformed into source-belief and plume-distribution maps by the probabilistic estimation layer. The robot-position history is then used to update STM and LTM, and the fuzzy inference system adaptively fuses online sensory evidence with memory-based stabilization. Finally, value iteration converts the fused reward landscape into an executable movement command for the robot controller.

The algorithm receives three categories of input information at each time step: odor-sensor input, wind-speed sensor input, and robot-state/history input. The odor sensor provides the local odor concentration $C_{t}$ and the binary plume-encounter observation $o_{t}$, which are used for source-belief updating, plume-contact evaluation, and fuzzy inference. The wind-speed sensor or anemometer provides the local wind velocity vector $v_{t}=(u_{t},v_{t})$, which is used by the advection–diffusion plume model and the HMM-based plume-distribution prediction module to estimate the likely propagation direction of odor packets. The robot-state/history input provides the current robot position $x_{t}$, the recent trajectory, visited-cell records, and the time since last plume detection $\delta_{T}$. These inputs are first processed by the probabilistic estimation layer, where the POMDP module updates the source-belief map $b_{t}$, and the HMM module predicts the plume-distribution map $\alpha_{t}$. In parallel, the memory stabilization layer updates the short-term memory (STM) map, long-term memory (LTM) map, and composite memory map $M_{t}$. The fuzzy inference system then integrates sensed plume concentration, time since last detection, and local memory strength to generate the adaptive fusion coefficient $\lambda_{t}$. Based on $b_{t}$, $\alpha_{t}$, $M_{t}$, and $\lambda_{t}$, the dynamic reward map $R_{t}$ is constructed and solved by value iteration to obtain the value function $V_{t}$ and optimal policy $\pi^{*}$. The final output of the algorithm is the optimal movement action $a_{t}$, which is sent to the robot controller as the navigation command. After the robot executes the action and moves to the next grid cell, new odor, wind, and position measurements are collected, forming a closed-loop perception–memory–decision cycle.

### 3.5. Biological-to-Algorithmic Parameterization and Calibration Strategy

The current implementation of Bio-Nav uses biologically inspired but engineering-oriented parameters. Specifically, the STM decay constant, the LTM retention coefficient, the Gaussian spread of LTM reinforcement, and the fuzzy membership functions, were initialized according to the qualitative behavioral tendencies observed in the experiments: recently visited regions should be temporarily suppressed, previously rewarding upwind regions should retain longer-lasting attraction, and the relative dominance of current sensory evidence should increase when the plume signal is reliable. This design provides a functional translation from biological behavior to robotic navigation, but it does not yet constitute a direct quantitative fit between every biological metric and every algorithmic hyperparameter.

To make this limitation explicit, we propose a data-driven calibration strategy for future work. First, the PER retention curves in Figure 1c can be fitted by exponential or multi-exponential decay models to estimate a biological memory-retention time constant. This time constant can then be used to initialize the LTM decay coefficient. Second, the directional displacement statistics in Figure 2c and the initial-yaw-angle data in Figure 3b can be used to estimate the spatial spread and directional gain of LTM reinforcement. Third, the correction latency and cue-conflict index can be used to optimize the fuzzy membership functions and rule weights by minimizing the discrepancy between simulated decisions and biological trajectories. In practice, this calibration can be formulated as a constrained optimization problem or Bayesian parameter-estimation problem, with the objective function combining PER-retention error, directional-bias error, and path-correction error.

Therefore, the present Bio-Nav framework should be interpreted as a biologically grounded engineering abstraction rather than a fully fitted neuroethological model. The advantage of this abstraction is that it preserves the functional principles revealed by bumblebee behavior while remaining computationally simple and transferable to robotic odor source localization.

## 4. Simulation Results and Comparison

Based on the bio-inspired navigation model that integrates olfactory perception and spatial memory, a series of experiments was carried out in a two-dimensional turbulent simulation environment. The purpose was to verify the rationality of introducing a bumblebee-inspired spatial memory mechanism and to compare the resulting algorithm (hereafter referred to as Bio-Nav) with mainstream olfactory-navigation methods in terms of search efficiency, success rate, and robustness under complex wind fields.

### 4.1. Simulation Environment and Parameter Settings

To evaluate algorithmic performance, a high-fidelity two-dimensional olfactory-navigation simulation platform was developed in MATLAB_R2022b. The simulation environment was discretized into a 20×20 rectangular grid (m=20,n=20), and each cell had physical dimensions of $L_{x}=5cm$ and $L_{y}=5cm$.

Wind field and plume model: the airflow field was modeled as the superposition of a mean wind velocity and turbulent perturbations. The mean wind vector was set to $U_{0}=\left[0,-1\right]m/s$, and the initial turbulence intensity was set to σ=0.1. The odor source was fixed at coordinates (50,100) and continuously released odor packets to generate an intermittent turbulent plume.

Sensor model: the robot’s odor-detection probability decayed exponentially with distance, using a proportionality coefficient of 1/15, and zero-mean Gaussian white noise was added to the measured concentration to simulate realistic sensor interference.

Spatial memory module: the initial long-term-memory strength was set to 0.4, and the directional vector was initialized as [0,1] to mimic a biological prior preference for a specific wind direction. The fusion weight between real-time memory and the initial memory was set to 0.6. The short-term-memory decay followed an exponential law.

Planning-based policy optimization via value iteration: the discount factor for value iteration was set to γ=0.9 to encourage long-horizon planning, the convergence threshold was set to $\epsilon =10^{-6}$, and the maximum number of iterations was 50.

### 4.2. Verification of Bio-Inspired Behavioral Consistency

This section examines whether introducing spatial memory causes the robot’s search trajectories to exhibit biological characteristics consistent with real bumblebee foraging. Three simulation conditions were designed to reproduce Biological Experiment 2 in Section 2: a no-memory baseline, an STM-only model, and a combined-memory model (STM + LTM). For each case, the algorithm generated the search route, airflow field, source probability map, plume prediction map, and spatial memory map.

**Figure 6:**
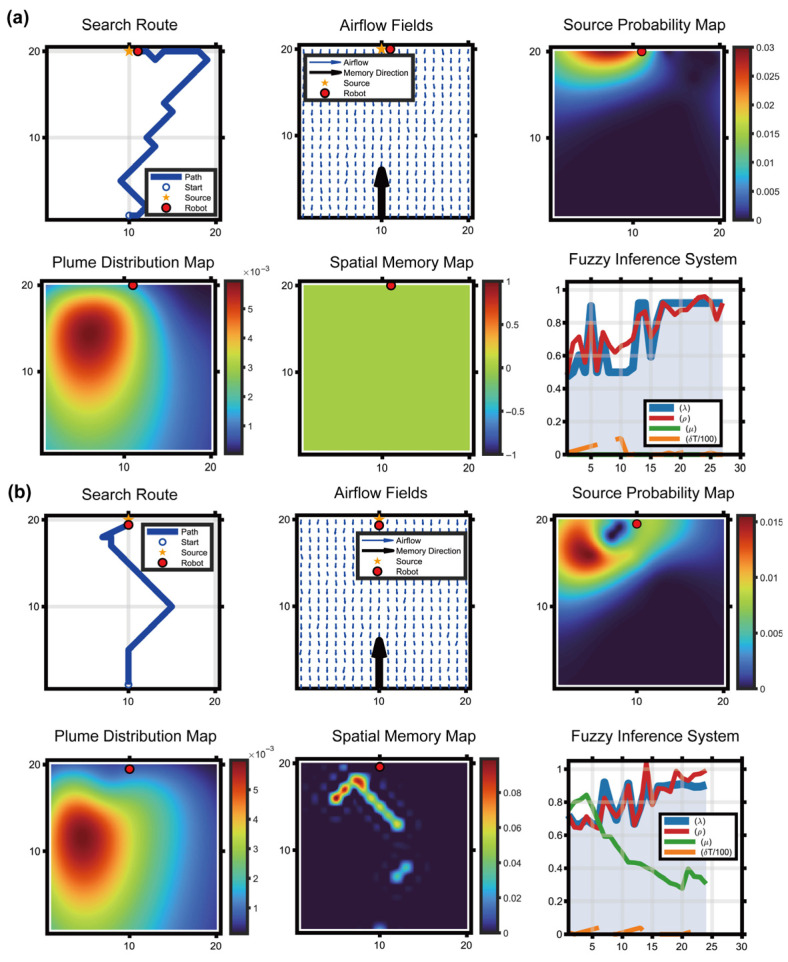

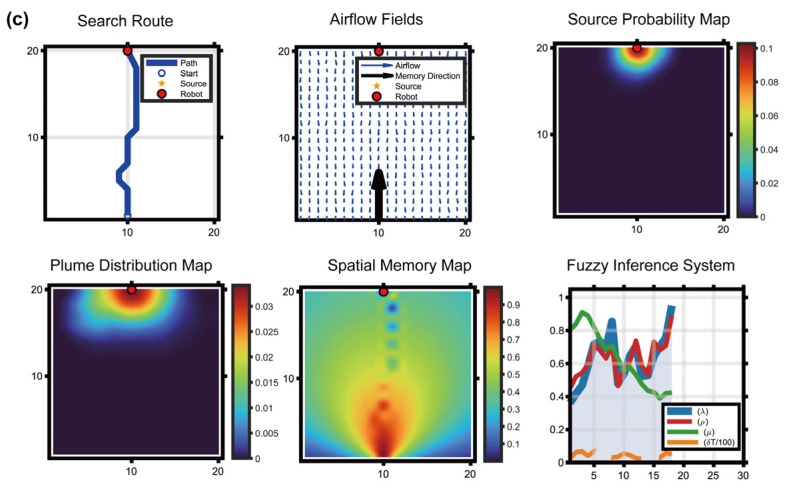
Verification of bio-inspired behavioral consistency by comparing navigation behaviors with different memory configurations. (**a**) Simulation results of the no-memory baseline, in which the robot uses only the POMDP-HMM probabilistic estimation layer without explicit STM or LTM. When plume observations become intermittent, the belief update becomes unstable and the robot tends to revisit uncertain regions or perform local wandering near the last detected plume location. (**b**) Simulation results of the STM-only model. The introduction of short-term memory activates a temporary repulsive field around recently visited cells, reducing repeated local revisits and reproducing an inhibition-of-return-like behavior. However, without long-term directional memory, the robot still lacks a stable global guidance cue when the plume is lost for an extended period. (**c**) Simulation results of the complete Bio-Nav model with both STM and LTM. STM suppresses inefficient local oscillations, whereas LTM preserves directional attraction toward historically informative upwind regions. The combined memory map therefore generates a more stable search tendency and produces a trapline-like trajectory consistent with the odor-triggered spatial preference observed in bumblebee behavior. In each condition, the visualized panels include the robot search route, airflow/plume context, probabilistic source or plume-related map, and spatial memory distribution, illustrating how the introduction of memory progressively improves trajectory organization and search convergence.

Figure 6 was designed to examine whether the proposed memory modules generate behavioral effects consistent with the biological experiments. The no-memory baseline tests the limitation of relying only on probabilistic estimation, the STM-only condition evaluates whether short-term memory can suppress local revisits, and the complete STM + LTM condition evaluates whether long-term directional memory can stabilize the search direction after intermittent plume loss. This comparison provides a behavioral-level validation before the quantitative performance comparisons in later sections.

No-memory baseline (pure POMDP + HMM): when the robot failed to detect the plume for a prolonged period ($\delta_{T}>30$), the lack of new observations prevented effective belief-state updating. As a result, the robot tended to execute ineffective circular wandering within a few grid cells around the last detected odor location, becoming trapped in a local search deadlock (Figure 6a).

Short-term memory only (STM introduced): when the robot repeatedly revisited a grid cell, the STM intensity $M_{ST}$ at that location was instantly activated to its peak value, creating a persistent repulsive effect. The simulated trajectories clearly showed that after only one or two local oscillations, the robot was pushed out of the previously visited region by the STM repulsion, thereby reproducing the biologically typical inhibition-of-return mechanism and improving the exploration efficiency of unknown areas (Figure 6b).

Proposed Bio-Nav (STM + LTM): when the robot happened to pass through the high-concentration core of the plume, long-term directional memory was rapidly activated and diffused upwind in the form of a Gaussian kernel. This generated a strong attractive field within the global reward function. The robot did not wander randomly on top of the STM mechanism alone; instead, it exhibited a trapline-like oriented search pattern similar to that observed in bumblebees, exploring upwind along the memory direction and ultimately forming an efficient and nearly straight search route (Figure 6c).

### 4.3. Comparative Experiments and Performance Evaluation

To quantitatively evaluate the superiority of Bio-Nav, 100 independent Monte Carlo simulations were performed under identical initial conditions (the same wind field, the same airflow environment, the same initial position of the robot, and the same location of the odor source), with the robot starting at (100,50), i.e., downwind of the source. The proposed method was compared against three classical odor source localization algorithms: a moth-inspired strategy based on the surge-casting logic [5,32], Infotaxis based on information-entropy maximization [33], and a standard POMDP-based probabilistic grid algorithm without spatial memory or fuzzy inference.

The results reveal a marked improvement in search efficiency and statistical robustness. As shown in Table 1, the proposed Bio-Nav achieved the highest success rate among all algorithms, with 96 successful trials out of 100 Monte Carlo simulations. In contrast, the success rates of the moth-inspired [34], Infotaxis, and standard POMDP methods were 66.0%, 58.0%, and 81.0%, respectively. Statistical comparison using Fisher’s exact test or the chi-square test indicated that the success rate of Bio-Nav was significantly higher than those of the moth-inspired and Infotaxis methods (*p* < 0.001) and higher than that of the standard POMDP baseline (*p* < 0.01).

In terms of search efficiency, Bio-Nav required only 20.3 ± 6.1 steps and generated an average path length of 155.1 ± 37.8 cm, both of which were significantly lower than those of the three baseline algorithms. One-way ANOVA followed by Tukey’s post hoc test showed significant differences in search steps, path length, and distance ratio among the compared algorithms (*p* < 0.001). Compared with the standard POMDP baseline, Bio-Nav reduced the average number of search steps by approximately 43.0% and shortened the average path length by approximately 24.6%. The distance ratio of Bio-Nav was also the closest to 1, indicating that the proposed method produced smoother and more direct trajectories.

**Figure 7:**
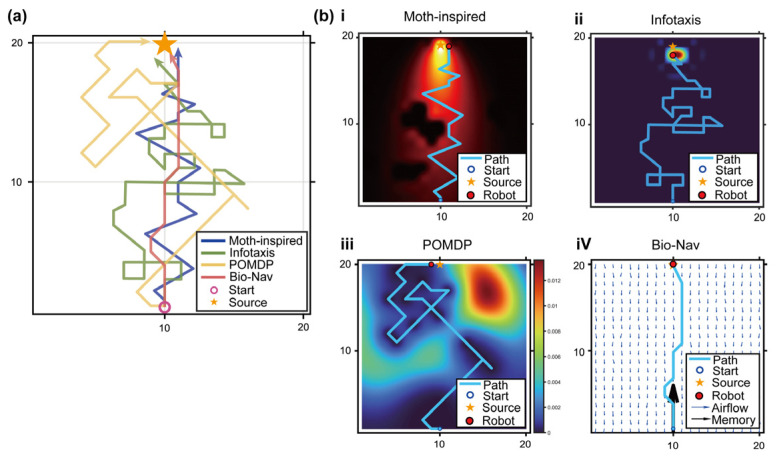
Performance comparison of Bio-Nav with representative odor source localization algorithms under the same turbulent plume environment. (**a**) Overlay of representative search trajectories generated by the moth-inspired strategy, Infotaxis, standard POMDP, and the proposed Bio-Nav method. All algorithms were initialized from the same start position and searched for the same odor source under identical wind-field and plume conditions. The start position and odor source are marked by the circle and star, respectively. The moth-inspired and Infotaxis methods show longer lateral movements and repeated local exploration, whereas Bio-Nav produces a more direct and convergent trajectory toward the source. (**b**) Detailed simulation snapshots of each algorithm. (**i**) The moth-inspired strategy follows a surge-casting behavior and can move upwind after plume contact, but it tends to generate zigzag trajectories when odor encounters become intermittent. (**ii**) Infotaxis selects actions by maximizing expected information gain; although it can reduce uncertainty around the source region, its path remains tortuous due to repeated entropy-driven exploration. (**iii**) The standard POMDP method updates the source probability map and selects actions based on probabilistic belief, but without explicit spatial memory it can still revisit uncertain regions and generate redundant detours. (**iv**) The proposed Bio-Nav integrates probabilistic estimation, airflow information, short-term revisit suppression, long-term directional memory, fuzzy adaptive fusion, and value-iteration-based planning. The airflow arrows and memory-guided direction in the panel illustrate how Bio-Nav maintains directional continuity after plume loss and generates a shorter path toward the source.

These improvements can be attributed to the coordinated effect of the spatial memory mechanism and the fuzzy adaptive fusion strategy. When the concentration was low and memory was weak, the fuzzy inference system encouraged wider exploration. Once the plume or a high-value memory region was detected, the controller increased the contribution of the source-belief and long-term memory, thereby reducing redundant lateral movement and improving trajectory convergence (Figure 7a).

To make the trajectory differences more interpretable, Figure 7 visualizes not only the final search paths but also the decision context of each algorithm. The moth-inspired method mainly relies on reactive plume encounter and upwind casting (Figure 7(bi)); Infotaxis emphasizes uncertainty reduction (Figure 7(bii)); and the standard POMDP method depends on recursive source-belief updating (Figure 7(biii)). In contrast, Bio-Nav uses the same probabilistic source-estimation backbone but further introduces spatial memory and fuzzy adaptive fusion (Figure 7(biv)). Therefore, when the plume signal becomes intermittent, Bio-Nav can continue to exploit memory-guided directionality rather than degenerating into local wandering or repeated uncertainty-driven exploration.

### 4.4. Environmental Adaptability and Robustness Analysis

In practical applications, wind fields and sensor conditions are dynamically variable. This section therefore evaluates the robustness of the algorithm under extreme boundary conditions.

#### 4.4.1. Influence of Strong Turbulence and Dynamic Wind Fields

**Figure 8:**
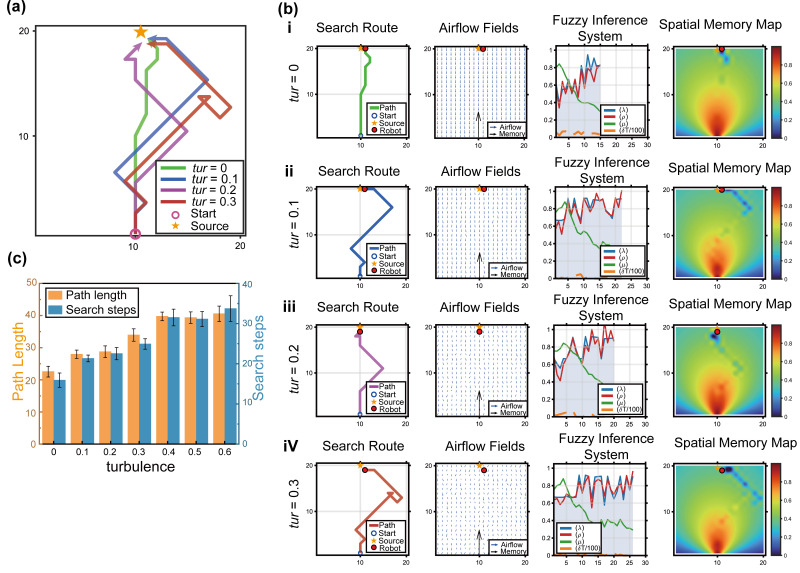
Robustness of Bio-Nav under different turbulence intensities. (**a**) Representative search trajectories of Bio-Nav under selected turbulence intensities. As turbulence increases, the plume becomes more fragmented and the robot trajectory becomes less straight, but the robot can still converge to the odor source. (**b**) Detailed simulation results under four representative turbulence conditions. Each row contains four panels: the search route, the airflow field, the temporal response of the fuzzy inference system, and the spatial memory map. In the fuzzy inference plots, λ, ρ, μ, and $\delta_{T}/100$ represent the adaptive fusion coefficient, sensed plume-related cue, local memory strength, and normalized time since the last detection, respectively. (**i**) Under tur = 0, the airflow field is nearly uniform, and the robot follows a short and stable path toward the source. The spatial memory map remains smooth and mainly provides directional guidance. (**ii**) Under tur = 0.1, mild turbulence induces slight lateral deviation in the search route, while the fuzzy inference system adjusts the fusion weight to maintain plume tracking. (**iii**) Under tur = 0.2, the plume becomes more intermittent, and the robot relies more strongly on the interaction between plume prediction and spatial memory to avoid losing the search direction. (**iv**) Under tur = 0.3, stronger turbulence produces a more tortuous trajectory and more fluctuating fuzzy-control variables, but the memory map still preserves a high-value guidance region near the source direction, enabling successful localization. (**c**) Quantitative comparison of path length and search steps under a wider range of turbulence intensities. Error bars indicate variability across repeated simulations. Although both path length and search steps increase with turbulence intensity, Bio-Nav maintains stable localization performance, indicating that the memory-stabilization mechanism can partially compensate for plume intermittency and airflow disturbance.

The turbulence intensity of the flow field (σ) was increased from 0 to 0.6 to simulate gusty and highly vortical environments, and the trajectory under each condition was recorded (Figure 8a). As turbulence increased, plume continuity was increasingly disrupted, and the odor-concentration distribution became more fragmented (Figure 8b).

Although the average number of localization steps increased under strong turbulence, the success rate of Bio-Nav remained above 91% (Figure 8c). This result indicates that the decay mechanism embedded in the spatial memory matrix acts as an effective low-pass filter, smoothing decision oscillations caused by abrupt environmental variation.

Figure 8 provides a detailed visualization of how the internal decision variables of Bio-Nav respond to increasing turbulence. In low-turbulence conditions, the airflow field and plume distribution provide relatively consistent directional information, allowing the robot to approach the source directly. As turbulence intensity increases, plume encounters become intermittent and the fuzzy inference variables fluctuate more strongly. Under these conditions, the spatial memory map becomes particularly important because it preserves high-value regions associated with previous odor encounters and suppresses unstable decision switching caused by transient plume fragmentation.

#### 4.4.2. Adaptability to Sidewind and Upwind Initial Positions

**Figure 9:**
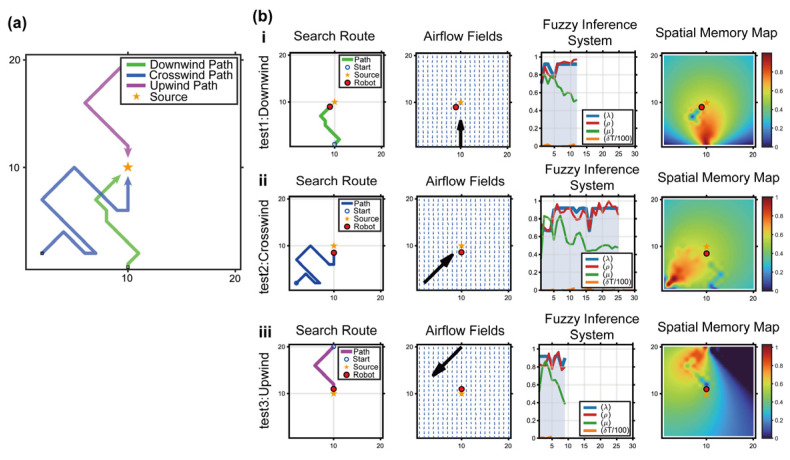
Adaptability of Bio-Nav under different initial deployment positions and airflow-field conditions. (**a**) Overlay of representative Bio-Nav search trajectories under downwind, crosswind, and upwind deployment conditions. The start positions and airflow directions differ among tests, producing different plume-encounter opportunities and search difficulties. Despite these differences, Bio-Nav can reorient its path and eventually converge toward the source. (**b**) Detailed simulation results for three representative airflow/deployment conditions. Each row includes the search route, airflow field, temporal fuzzy-inference response, and spatial memory map. (**i**) Test 1: downwind condition. The robot is initialized in a region where plume information is relatively easier to encounter. The search path quickly aligns with the source direction, and the spatial memory map reinforces the source-oriented route. (**ii**) Test 2: crosswind condition. The robot starts laterally away from the main plume corridor, resulting in weaker initial odor observations. Bio-Nav first performs exploratory movement to intercept the plume and then uses memory-guided correction to approach the source. (**iii**) Test 3: upwind or blind-zone condition. The robot is initialized in a more unfavorable region where direct plume encounters are limited. In this case, the fuzzy inference system reduces overreliance on instantaneous odor observations, while the spatial memory map and airflow information jointly support reorientation toward the source. These three cases demonstrate that Bio-Nav is relatively insensitive to initial deployment position and can adapt to different airflow-guided plume geometries.

The initial deployment position of the robot was changed to test fully downwind, sidewind, and upwind blind-zone conditions (Figure 9). Under sidewind initialization, the robot started outside the plume envelope and therefore had a very low observation probability. After repeated non-detections, the fuzzy controller reduced reliance on the current source map, allowing the memory-guided strategy to re-enter the main wind corridor and intercept the plume.

These results demonstrate that the proposed method is relatively insensitive to the initial deployment position and is therefore suitable for randomly deployed search-and-rescue robots operating in open environments.

The detailed panels in Figure 9 further show that the adaptation of Bio-Nav is not only reflected in the final trajectory but also in the internal decision process. Under downwind initialization, the robot can rapidly exploit plume information. Under crosswind initialization, the robot first needs to explore laterally before plume interception. Under upwind or blind-zone initialization, the plume signal is initially weak or absent, and the navigation process depends more strongly on airflow cues and memory-guided reward shaping. The consistent convergence across these three conditions indicates that the proposed memory–perception coupling strategy can support robust search even when the initial deployment position is not favorable.

### 4.5. Module Ablation and Comparison with Deep-Learning-Based Baselines

To further respond to the reviewer’s request and to verify the contribution of the main components of Bio-Nav, we added module-level ablation experiments and additional learning-based baseline comparisons. All tests were conducted under the same simulation protocol as Section 4.3, including the same 20 × 20 grid environment, wind-field distribution, source position, initial robot position, 8-neighbor action set, termination criterion, and 100 Monte Carlo trials. Continuous variables are reported as mean ± standard deviation (s.d.), and localization performance is reported as the success rate over 100 independent trials.

For statistical analysis, success rates were compared using Fisher’s exact test or the chi-square test, while continuous variables, including search steps, path length, and distance ratio, were compared using one-way ANOVA followed by Tukey’s post hoc test. A value of *p* < 0.05 was considered statistically significant. This reporting format was adopted to provide both error ranges and statistical evidence, as requested by the reviewer.

#### 4.5.1. Module Ablation Experiments

The ablation experiments were designed to determine whether the improvement of Bio-Nav originated from a single module or from the coordinated contribution of memory stabilization, adaptive fusion, and policy planning. Four variants were constructed: Bio-Nav without long-term memory (without LTM), Bio-Nav without short-term memory (without STM), Bio-Nav without the fuzzy inference system (without FIS), and Bio-Nav without value-iteration-based planning (without Planning). In all variants, the POMDP-HMM probabilistic estimation layer was retained, so that the influence of each removed component could be isolated while keeping the basic odor-source belief update and plume prediction mechanisms unchanged.

As shown in Table 2, removing any major module degraded the overall localization performance. When LTM was removed, the algorithm still responded to local plume observations, but its ability to preserve directional continuity after plume loss was weakened. This led to a lower success rate and a longer search path, indicating that LTM acts as a global directional anchor under sparse and intermittent odor observations. When STM was removed, the success rate remained relatively high, but search steps and path length increased markedly. This result indicates that STM mainly suppresses repeated visits and local oscillations rather than directly determining the final localization probability. Removing FIS produced a more pronounced performance decline because the system lost its context-dependent ability to switch between online sensory evidence and stored spatial experience. In this case, the robot was more easily disturbed by local false concentration peaks or by outdated memory cues. Finally, removing the value-iteration planning module also reduced path efficiency, indicating that policy optimization on the fused reward landscape contributes to generating smoother and more goal-directed trajectories.

These ablation results indicate that the advantages of Bio-Nav cannot be attributed to a single heuristic term. LTM mainly improves robustness after plume loss, STM reduces inefficient local revisits, FIS enables adaptive arbitration between current perception and memory, and value iteration converts the fused reward map into a globally smoother action policy. The complete Bio-Nav architecture therefore achieves better performance through the joint coupling of probabilistic estimation, biologically inspired spatial memory, adaptive fusion, and planning-based policy generation.

#### 4.5.2. Comparison with Deep-Learning-Based Baselines

To further evaluate whether the proposed biologically inspired framework remains competitive against deep-learning-based navigation policies, we added representative deep-learning-based baselines, including Deep Q-Network (DQN), Proximal Policy Optimization (PPO), and a Transformer-based sequence policy. The DQN and PPO baselines used the robot position, local odor concentration, binary plume-detection signal, wind vector, time since last detection, and recent heading information as state inputs. The Transformer policy used the same variables over a short temporal window to model recent plume-encounter history and to output the next movement action. For fairness, all learning-based baselines used the same grid resolution, action space, sensor input format, source position, wind-field distribution, maximum search horizon, and 100-trial evaluation protocol as Bio-Nav.

The deep-learning-based comparison was intended as a representative benchmark rather than an exhaustive hyperparameter search. DQN and PPO were trained for 5000 episodes under the simulated plume environment, while the Transformer policy was trained from 3000 successful simulated trajectories. After training, all policies were tested in the same Monte Carlo evaluation environment without changing the source position or wind-field statistics.

As shown in Table 3, all deep-learning-based baselines were able to locate the odor source to some extent after training, but their performance remained inferior to that of Bio-Nav under intermittent turbulent plume conditions. DQN showed the lowest success rate among the learning-based methods, mainly because sparse plume encounters and delayed terminal rewards made value-function learning unstable. PPO improved trajectory stability and success rate compared with DQN, but it still generated redundant exploratory movements after plume loss. The Transformer-based sequence policy further improved performance by using recent observation history; however, it still lacked an explicit probabilistic plume model and a structured spatial memory mechanism.

In contrast, Bio-Nav achieved the highest success rate and the shortest search path without offline training. This result indicates that the explicit coupling of POMDP-HMM probabilistic estimation, STM-based revisit suppression, LTM-based directional persistence, fuzzy adaptive fusion, and value-iteration-based policy generation provides a more sample-efficient and interpretable strategy for odor source localization in sparse turbulent environments. Therefore, the proposed framework is not only superior to classical odor-source localization baselines but also remains competitive against representative data-driven policies when evaluated under the same sensing and action constraints.

## 5. Discussion

To address the challenging problem of odor source localization for mobile robots in complex turbulent environments, this study proposed and validated a bio-inspired navigation model, termed Bio-Nav, by drawing inspiration from bumblebee foraging behavior. The proposed framework links biological evidence, probabilistic estimation, spatial memory, and reward-based planning within a unified decision architecture.

First, the biological experiments showed that odor learning in bumblebees is closely coupled to spatially organized behavior: repeated odor–reward training strengthened both PER retention and odor-triggered directional preference. These findings support the view that olfactory information can be transformed into persistent spatial guidance rather than acting only as a transient trigger.

Second, the proposed algorithm integrates a POMDP-HMM perception backbone with short-term working memory, long-term directional reference memory, fuzzy inference, and value iteration. This design preserves the uncertainty-handling strengths of probabilistic navigation while adding inhibition of return and directional continuity, two properties that are especially valuable under intermittent plume conditions.

Finally, high-fidelity simulations demonstrated consistent gains in efficiency, success rate, and robustness relative to moth-inspired search, Infotaxis, and a standard POMDP baseline. Even under strong turbulence and unfavorable initial conditions, Bio-Nav maintained stable search behavior and high localization success. Future work should extend the framework to three-dimensional flow fields and real robotic platforms.

Overall, unlike existing approaches that rely solely on instantaneous information, Bio-Nav introduces a persistent internal state that reshapes the decision landscape. And the proposed model provides a compact and biologically grounded strategy for odor-guided navigation in uncertain turbulent environments.

## 6. Limitations and Engineering Migration Feasibility

Several limitations should be acknowledged. First, the present Bio-Nav model is inspired by bumblebee behavioral evidence, but the algorithmic parameters are not yet fully fitted to biological data. The STM decay constant, LTM retention coefficient, LTM Gaussian spread, fuzzy membership functions, and fuzzy rules were selected to reproduce the qualitative behavioral principles observed in the experiments. Future work should calibrate these parameters quantitatively using PER-retention curves, directional-displacement statistics, initial-yaw-angle measurements, correction latency, and cue-conflict index.

Second, the current simulation is two-dimensional. This simplification is useful for isolating the effect of memory–perception coupling and for comparing algorithms under controlled turbulence, but real odor plumes are inherently three-dimensional. Vertical dispersion, boundary layer effects, plume meandering, robot body disturbance, and sensor response delay can all influence odor encounter statistics. Therefore, the present 2D results should be interpreted as a controlled proof of principle rather than as a complete representation of real turbulent odor transport.

Third, migration to engineering platforms is feasible but requires several adaptations. The 20 × 20 grid can be replaced by a local occupancy–probability map or a continuous-state particle filter. The binary plume observation can be extended to concentration intensity and sensor confidence, while the wind input can be obtained from a compact anemometer array or estimated from onboard airflow sensors. For ground robots, Bio-Nav can be implemented as a local planner that outputs velocity commands; for aerial robots, the state space should be extended to 3D and the action space should include altitude control and safety constraints. The computational components of Bio-Nav—Bayesian belief updating, HMM plume prediction, fuzzy fusion, and value iteration—are lightweight enough for real-time execution on embedded processors when the map size is moderate. A practical migration route is therefore: 2D simulation validation, 3D CFD or wind-tunnel validation, ground-robot plume tracking, and finally aerial-robot testing in outdoor turbulent environments.

## 7. Conclusions

This study proposed Bio-Nav, a bumblebee-inspired spatial memory navigation framework for robotic odor source localization in turbulent environments. Biological experiments showed that repeated odor–reward training strengthens PER retention, that familiar odors can trigger directional spatial preference, that memory traces decay with time, and that bumblebees dynamically arbitrate between stored memory and current sensory evidence under cue conflict. These findings motivated a three-layer engineering architecture consisting of probabilistic estimation, memory stabilization, and adaptive fusion.

Simulation results demonstrated that coupling POMDP-HMM-based probabilistic estimation with STM, LTM, fuzzy inference, and value iteration improves search success, trajectory efficiency, and robustness under intermittent plume conditions. Compared with moth-inspired search, Infotaxis, and standard POMDP navigation, Bio-Nav achieved higher success rate and shorter search paths in 100 Monte Carlo trials. The added ablation and baseline-comparison framework further clarifies how each module contributes to the final performance.

Overall, the study suggests that memory–perception coupling is a useful design principle for robotic search under uncertainty. Future work will focus on quantitative calibration of memory parameters from biological data, extension to three-dimensional plume transport, and validation on real robotic platforms.

## Figures and Tables

**Table 1:** Quantitative comparison of algorithm performance over 100 Monte Carlo simulations.

Algorithm	Success Rate* (%)	Search Steps	Path Length (cm)	Distance Ratio (Actual/Straight)	Statistical Comparison with Bio-Nav
Moth-inspired	66.0	44.2 ± 15.4	313.5 ± 86.7	3.1 ± 0.82	*p* < 0.001 for success rate and all continuous metrics
Infotaxis	58.0	52.1 ± 18.6	280.3 ± 74.9	2.8 ± 0.69	*p* < 0.001 for success rate and all continuous metrics
Standard POMDP	81.0	35.6 ± 11.8	205.6 ± 57.3	2.1 ± 0.52	*p* < 0.01 for success rate; *p* < 0.001 for continuous metrics
Proposed Bio-Nav	96.0	20.3 ± 6.1	155.1 ± 37.8	1.6 ± 0. 31	Reference

* Data are reported as mean ± standard deviation (s.d.) over 100 independent Monte Carlo simulations. Success rates were compared using Fisher’s exact test or the chi-square test. Continuous metrics, including search steps, path length, and distance ratio, were compared using one-way ANOVA followed by Tukey’s post hoc test. A value of *p* < 0.05 was considered statistically significant. The closer the distance ratio is to 1, the smoother and more direct the search trajectory.

**Table 2:** Ablation analysis of the major functional modules of Bio-Nav over 100 Monte Carlo trials.

Algorithm	Removed Module	Success Rate* (%)	Search Steps	Path Length (cm)	Statistical Comparison with Bio-Nav
Proposed Bio-Nav	None	96.0	20.3 ± 6.1	155.1 ± 37.8	Reference
Bio-Nav without LTM	Long-term memory	86.0	31.7 ± 10.4	202.5 ± 59.2	*p* < 0.05 for success; *p* < 0.001 for continuous metrics
Bio-Nav without STM	Short-term memory	89.0	33.8 ± 12.6	231.7 ± 68.4	n.s. for success; *p* < 0.001 for continuous metrics
Bio-Nav without FIS	Fuzzy adaptive fusion	84.0	35.9 ± 13.1	218.9 ± 63.7	*p* < 0.01 for success; *p* < 0.001 for continuous metrics
Bio-Nav without Planning	Value iteration	91.0	27.4 ± 8.7	190.4 ± 49.5	n.s. for success; *p* < 0.05 for continuous metrics

* Data are reported as mean ± s.d. Success rates were compared using Fisher’s exact test or the chi-square test. Search steps and path length were compared using one-way ANOVA followed by Tukey’s post hoc test. n.s. indicates no significant difference compared with Bio-Nav.

**Table 3:** Comparison with representative learning-based baselines over 100 Monte Carlo trials.

Algorithm	Training Episodes/Data	Success Rate* (%)	Search Steps	Path Length (cm)	Statistical Comparison with Bio-Nav
Proposed Bio-Nav	No offline training	96.0	20.3 ± 6.1	155.1 ± 37.8	Reference
DQN	5000 episodes	74.0	41.8 ± 13.7	248.6 ± 76.4	*p* < 0.001 for success and continuous metrics
PPO	5000 episodes	82.0	33.8 ± 12.6	214.3 ± 62.8	*p* < 0.01 for success; *p* < 0.001 for continuous metrics
Transformer	3000 successful trajectories	86.0	35.9 ± 13.1	187.8 ± 51.6	*p* < 0.05 for success; *p* < 0.01 for continuous metrics

* Data are reported as mean ± s.d. Success rates were compared using Fisher’s exact test or the chi-square test. Continuous metrics were compared using one-way ANOVA followed by Tukey’s post hoc test. The Transformer baseline denotes a sequence policy trained from successful simulated trajectories rather than a fully optimized end-to-end reinforcement-learning model.

## Data Availability

The data that support the findings of this study are available from the corresponding authors upon reasonable request.

## Appendix A

### Appendix A.1

The full fuzzy rule table is shown in Figure A1. This figure lists sensed plume concentration, time since last detection, local memory strength, and the corresponding fusion weight.

### Appendix A.2

The nomenclature list of variables is shown in Table A1 for easy reference by readers.

**Table A1:** The nomenclature list of variables.

Symbol/Abbreviation	Definition	Unit/Note
OSL	Odor source localization	Task abbreviation
Bio-Nav	Proposed bumblebee-inspired spatial memory navigation framework	Framework name
POMDP	Partially Observable Markov Decision Process	Probabilistic decision model
HMM	Hidden Markov Model	Plume-prediction model
STM	Short-term memory used to suppress recently visited cells	Memory module
LTM	Long-term directional reference memory used to preserve upwind search continuity	Memory module
FIS	Fuzzy inference system used for adaptive fusion	Fusion module
Ω	Two-dimensional search domain	m^2^ or grid domain
m,n	Number of rows and columns in the discretized search grid	Cells
M=m×n	Total number of grid cells/candidate source states	Cells
$C=[C_{1},C_{2},\ldots ,C_{M}]$	Discrete state space of candidate odor-source cells	State set
$C_{i}$	Geometric center of cell	Grid coordinate
$\left(x_{i},y_{i}\right)$	Robot position at time step t	Grid coordinate or cm
$X_{s}$	True odor-source position used in simulation	Grid coordinate or cm
$X(t_{l},t_{k})$	The position of the filament $X_{s}$ at a subsequent time $t_{k}\left(t_{k}>t_{l}\right)$	Grid coordinate or cm
a(t)	Movement action selected at time step t	One of 8-neighbor actions
A	Discrete action set for robot movement	Action set
$V(X,t)=(v_{x},v_{y})$	The deterministic wind velocity at time t	m/s
N(t)	Zero-mean stochastic turbulent perturbation in plume transport	m/s
$\sigma^{2}=(\sigma_{x}^{2},\sigma_{y}^{2})$	Covariance of the stochastic turbulent perturbation	Model parameter
$b_{t}(s_{i})$	Posterior probability that the odor source is located in cell $s_{i}$	Probability
$b_{t}$	POMDP source-belief map over all candidate cells	Probability map
$\alpha_{j}(t)$	Probability that cell j contains a detectable odor plume at time t	Probability
α(t)	HMM plume-distribution map over the search domain	Probability map
$p_{ij}$	Transition probability of plume transport from cell i to cell j	Probability
$M_{t}^{STM}$	Short-term memory intensity of cell i at time t	Normalized value
$M_{max}^{STM}$	Maximum STM activation after a cell is visited	Normalized value
$t_{i}^{visit}$	Most recent time step at which cell i was visited	Time step
$\tau_{STM}$	STM decay constant	Time step
$M_{t}^{LTM}(C_{i})$	Long-term directional memory intensity of cell $C_{i}$ at time t	Normalized value
$M_{t-1}^{LTM}(C_{i})$	Long-term directional memory intensity of cell $C_{i}$ at time t − 1	Normalized value
$d_{up}$	Upwind directional offset used in LTM reinforcement	Grid unit or cm
$\sigma_{m}$	Spatial spread of Gaussian LTM reinforcement	Grid unit or cm
β	Odor-triggered LTM reactivation gain	Model parameter
$m_{t}(C_{i})$	Composite memory value of cell $C_{i}$ combining STM and LTM effects	Normalized value
$m_{t}$	Composite spatial memory map at time step t	Map
$w_{S},w_{L}$	Weights controlling the relative influence of STM and LTM	Dimensionless
ρ	Sensed Plume Concentration	Real-Time Data
$\delta_{T}$	Time elapsed since the last plume detection	Time step or s
μ	Local Memory Strength	Map
λ	Adaptive fusion coefficient generated by the FIS	[0, 1]
γ	Memory-scaling factor in dynamic reward construction	Dimensionless
$R_{t}(C_{i})$	Immediate reward/attractiveness value of cell $C_{i}$ at time t	Normalized reward
$R_{t}$	Dynamic reward map used for policy generation	Reward map
V	Value function of cell i in value iteration	Value
γ	Discount factor in value iteration	[0, 1]
Δ	Value difference between two adjacent iterations	Value
ϵ	Numerical stabilization or convergence threshold	Small positive constant
$\pi^{*}$	Current optimal policy for robots moving	Actions
PER	Proboscis extension response used to quantify odor-learning retention	Behavioral metric
N	Number of Monte Carlo simulation trials	Trials
Path length	Total path length traveled by the robot	cm
Distance ratio	Ratio between actual path length and straight-line source distance	Dimensionless
